# Prognostic biomarker IL17A correlated with immune infiltrates in head and neck cancer

**DOI:** 10.1186/s12957-022-02703-1

**Published:** 2022-07-28

**Authors:** Meng Yu, Xing Xing Qian, Guang Li, Zexing Cheng, Zhijie Lin

**Affiliations:** 1 Department of Otorhinolaryngology-Head and Neck Surgery, The Affiliated Hospital of Yangzhou University, Yangzhou University, Yangzhou, 225009 People’s Republic of China; 2 Health Management Center, Affiliated Hospital of Yangzhou University, Yangzhou University, Yangzhou, 225009 People’s Republic of China; 3 Department of Immunology, Institute of Translational Medicine, School of Medicine, Yangzhou University, Yangzhou, 225009 People’s Republic of China; 4 Jiangsu Key Laboratory of Integrated Traditional Chinese and Western Medicine for Prevention and Treatment of Senile Diseases, Yangzhou, 225009 People’s Republic of China

**Keywords:** IL-17A, Head and neck cancer, TCGA, B cells, Gene expression

## Abstract

**Background:**

The underlying functions and mechanisms of the Th17 pathway in Head and neck squamous cell carcinoma (HNSCC) progression and tumor immunology are still unclear. We investigated the correlation between IL17A expression and certain clinical parameters, tumor-infiltrating immune cells (TIICs) in TCGA HNSCC samples.

**Methods:**

HNSCC files from the TCGA database were analyzed to obtain data on immune system infiltrates, gene expression, and related clinical information. R (Version 3.6.3) software, GEPIA, and TIMER online analysis tools were used to profile the relationship between the expression of IL17A and the prognosis, clinical stages, survival status and immune cell tumor-infiltrating levels of HNSCC patients. GEPIA and TIMER online analysis tools were used to verify the data.

**Results:**

The expression of IL17A was significantly decreased in tumor tissues from HNSCC. IL17A expression was associated with M, N stage, lymphovascular invasion, and patients OS event. GSEA revealed that IL17A was closely related to humoral immune response, T cells response, and cytokine signal. TCGA database and TIMER online analysis indicated that the B cells and T cells levels were correlated with IL17A. The correlation between IL17A expression and correlated genes was analyzed.

**Conclusions:**

IL-17A plays a key role in HNSCC. The levels of IL17A are important values for the determination of the occurrence and development of the HNSCC. The IL17A and correlated genes may be potential immunotherapeutic targets for HNSCC.

**Supplementary Information:**

The online version contains supplementary material available at 10.1186/s12957-022-02703-1.

## Background

Head and neck squamous cell carcinoma (HNSCC) is a common malignant tumor. It is collectively referred to as squamous cell carcinomas that line the mucosal surfaces of the head and neck. Head and neck cancer can form in the lip, oral cavity, pharynx (nasopharynx, oropharynx, and hypopharynx), paranasal sinuses and nasal cavity, larynx, and salivary glands [1, 2]. In the USA, these tumors are estimated 66,660 new cases and 14,620 deaths in 2021 [3]. Worldwide, GLOBOCAN 2020 estimates of incidence and mortality, there were approximately 830,000 new cases of tumors arising in the lip, oral cavity (377,713), larynx (184,615), nasopharynx (133,354), hypopharynx (84,254), and salivary glands (53,583), which accounted for 4.4% of the new global cancer cases. Approximately 420,000 patients died of these tumors, which accounted for 3.4% of global cancer-related deaths [4].

The main reason for this high mortality is treatment failure caused by resistance to conventional chemotherapy and local recurrence, cervical node metastasis in patients with advanced HNSCC. The survival rate among advanced HNSCC patients is only 34.9% and the effectively multidisciplinary treatment for HNSCC is still limited [5, 6]. Emerging studies from large HNSCC patient cohorts have been carried out to find predictive biomarkers to help clinicians make accurate early diagnoses, predict clinical outcomes and provide a reference for individualization of immunotherapy for HNSCC patients. High-throughput gene expression analyses were used to identify gene biomarkers related to the HNSCC prognosis [7, 8].

The human immune system is responsible for recognizing self-versus non-self and protecting the body from diseases of exogenous and endogenous origins. The immune system has been reported to be closely associated with the initiation and progression of cancer and immunotherapy has become the main strategy of cancer therapy [9]. Immunotherapy drugs that aim to modulate anti-tumor immune response have been approved to treat HNSCC cancer. However, immunotherapy is not yet as widely used as surgery, chemotherapy, or radiation therapy, and the more efficacy immunotherapy strategy for HNSCC is still under exploration [10]. Recent studies revealed that the IL-17 and its receptor signaling pathway are involved with the tumorigenesis and progression of several cancers. Targeting the IL-17 pathway may be a new strategy in the prevention and immunotherapy of cancer [11, 12]. However, the underlying functions and mechanisms of IL17A in HNSCC progression and immune cell tumor-infiltration are still unclear.

In the current study, using The Cancer Genome Atlas Program (TCGA) database and R (Version 3.6.3), Gene Expression Profiling Interactive Analysis (GEPIA) and Tumor Immune Estimation Resource (TIMER) online analysis tools, we evaluated the correlation between IL17A expression and certain clinical parameters in HNSCC samples. The Gene Set Enrichment Analysis (GSEA), Gene Ontology (GO) and Kyoto Encyclopedia of Genes and Genomes (KEGG) analyses were performed to evaluate the function of IL17A in HNSCC development. The correlation between IL17A and tumor-infiltrating immune cells (TIICs) was also determined. Our data present an elaborated analysis of the role of IL-17A in HNSCC development and contribute to understanding the molecular mechanisms underlying HNSCC.

## Material and methods

### Evidence from the TCGA database

We utilized the TCGA database (https://portal.gdc.cancer.gov) for HNSCC to obtain data on immune system infiltrates, gene expression (Level 3 HTSeq-FPKM), and related clinical information (contained 44 normal and 502 tumor samples). Differential gene expression analysis was performed between the high and low IL17A expression group of HNSCC [13]. The TNM stage classification followed the 8th Edition TNM Classification for HNSCC of the American Joint Committee on Cancer (AJCC). Kaplan-Meier (K-M) survival analysis was performed to analyze the relationship between the expression of IL17A and the survival status of HNSCC patients [14]. Five hundred two HNSCC patients were divided into two groups according to levels of IL17A expression, and the clinical characteristics are shown in Supplementary Table 1.

### Gene set enrichment analysis

GSEA (https://www.gsea-msigdb.org/gsea/msigdb/collections.jsp) was used to normalize RNA-Seq data obtained from TCGA [15]. The GO terms and the KEGG pathways enrichment analysis were performed using the R package cluster profiler for genes [16] to investigate possible biological functions of IL17A (including GO terms in three categories, biological processes, molecular function, cellular component, and KEGG pathway terms). To be deemed statistically significant, enrichment results had to satisfy two conditions, a false discovery rate (FDR) < 0.050 and a nominal *p* value < 0.050.

### Tumor-infiltration levels of immune cells analysis

TIMER is an integrative resource for investigating the molecular characterization of tumor-immune interactions across various cancer types (https://cistrome.shinyapps.io/timer/) [17, 18]. TIMER utilizes a deconvolution statistical method to deduce the abundance of six tumor-infiltrating immune cells, including B cells, CD4^+^ T cells, CD8^+^ T cells, macrophages, neutrophils and DCs from The Cancer Genome Atlas (TCGA). The gene module was used to analyze IL17A expression in different types of cancer and the correlation of IL17A expression with the abundance of immune infiltrates. The correlation module was used to explore the relationships between IL17A expression and gene markers of tumor infiltrates. The GEPIA (http://gepia.cancer-pku.cn/?from=timeline&isappinstalled=0) was used to further validate the significantly correlated genes in TIMER. The correlation between IL17A expression and markers of 24 immune cell types described previously [19] was validated using gene set variation analysis [20].

### Statistical analysis

The univariate and multivariate models of the Cox analysis were used to calculate the 95% CI and HR. Univariate survival analysis was used to compare several clinical characteristics with survival rates. The logistic regression analysis was used to evaluate correlations between clinical characteristics and IL17A expression. All statistical analyses were conducted using R (Version 3.6.3). Statistical significance was indicated as *p < 0.05; **p < 0.01; and ***p < 0.005.

## Results

### Decreased IL17A mRNA expression in HNSCC tumor

**Fig. 1:**
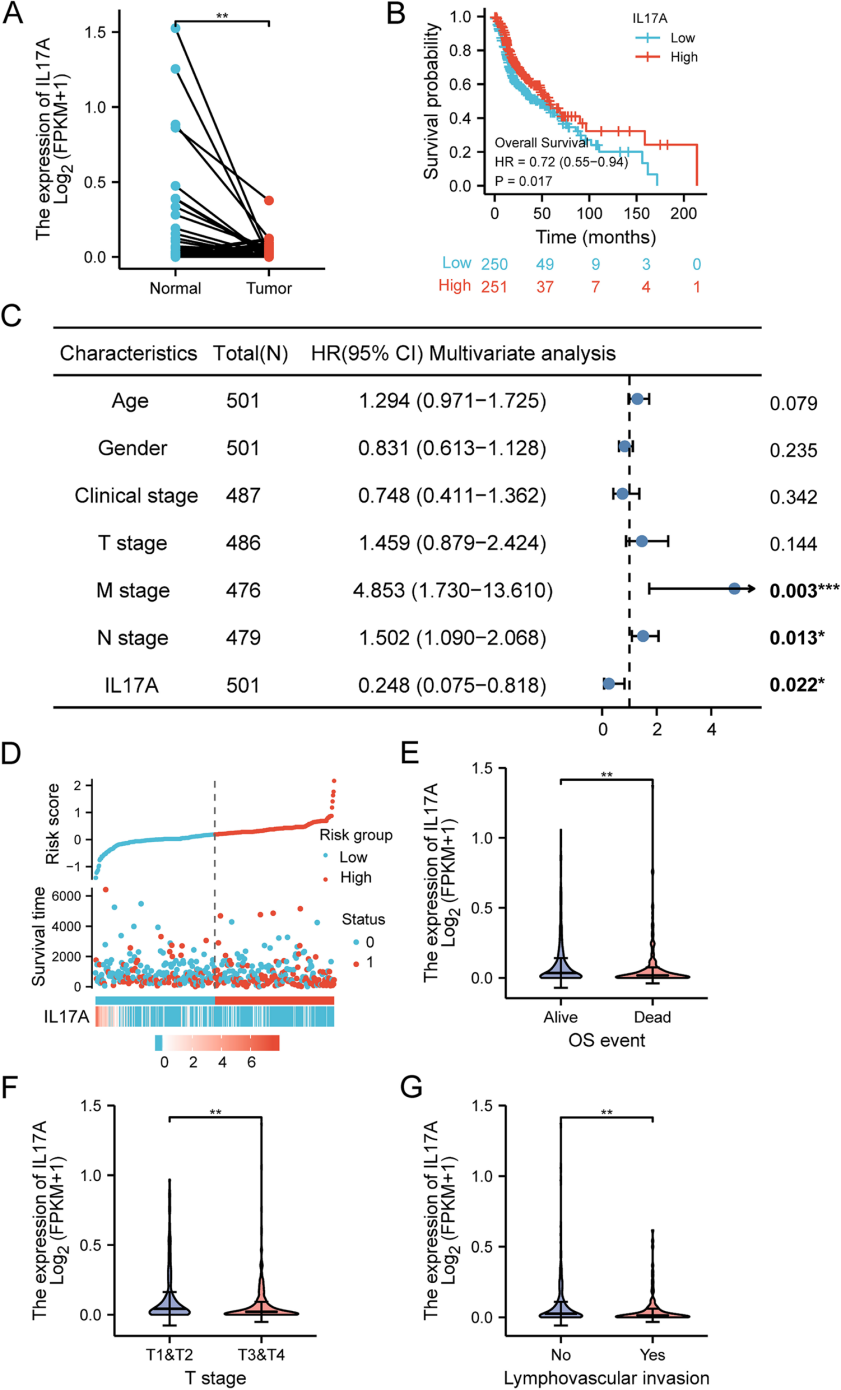
The expression of IL17A between normal and HNSCC tumor tissues (**A**). Kaplan–Meier survival curves comparing the high and low expression of IL17A in HNSCC patients (**B**). Multivariate Cox analysis of IL17A expression and other clinicopathological variables (**C**). IL17A expression distribution and survival status (D, 0, Alive; 1, dead). Expression of IL17A significantly correlated with over survival status (**E**), clinical T stage (**F**) and lymphovascular invasion (**G**). *p < 0.05, **p < 0.01, ***p < 0.005

To evaluate the correlation between IL17A and HNSCC, we used the TCGA database to identify differences in the levels of IL17A mRNA in normal and tumor tissues. A total of 502 tumors and 44 normal samples were transformed and converted into counts data. The expression of IL17A in normal and HNSCC samples was plotted (Fig. 1A and Supplementary Figure 1A). The unpaired data revealed that the IL17A expression was similar in normal and tumor tissues (Supplementary Figure 1A, *p* = 0.38), but paired analysis showed significantly decreased IL17A expression in tumor tissues compared with that in paired normal samples (Fig. 1A, *p* = 0.0055).

We investigated the correlations between the levels of IL17A expression and overall survival in HNSCC patients. As revealed in Fig. 1B, IL17A expression was significantly correlated with the prognosis of HNSCC (HR = 0.72, 95% CL 0.55–0.94, *p* = 0.017). The data were verified in GEPIA and UCSC Xena online databases (Supplementary Figure 1B, C). Next, we analyzed the correlation between overall survival and multivariable characteristics in HNSCC. Univariate analysis of correlation revealed three characteristics, M stage (HR = 4.745, *p* = 0.002), N stage (HR = 1.384, *p* = 0.026), and IL17A expression (HR = 0.250, *p* = 0.016) are significantly correlated with OS (Table 1). Using multivariate analysis, the data revealed that IL17A expression (HR = 0.284, *p* = 0.022) is an independent factor for prognosis (Fig. 1C and Table 1). The distribution of IL17A expression, survival status and risk score of patients with HNSCC were analyzed, as shown in Fig. 1D, group with low-risk score tend to have higher levels of IL17A expression and more survival compared with the high-risk group.

**Table 1:** Correlation between overall survival and multivariable characteristics in HNSCC patients

Characteristics	Total (*N*)	Univariate analysis		Multivariate analysis	
		Hazard ratio (95% CI)	*P* value	Hazard ratio (95% CI)	*P* value
Age	501	1.252 (0.956–1.639)	0.102	1.294 (0.971–1.725)	0.079
Gender	501	0.764 (0.574–1.018)	0.066	0.831 (0.613–1.128)	0.235
Clinical stage	487	1.217 (0.878–1.688)	0.238	0.748 (0.411–1.362)	0.342
T stage	486	1.245 (0.932–1.661)	0.137	1.459 (0.879–2.424)	0.144
M stage	476	4.745 (1.748–12.883)	0.002***	4.853 (1.730–13.610)	0.003***
N stage	479	1.384 (1.040–1.842)	0.026*	1.502 (1.090–2.068)	0.013*
IL17A	501	0.250 (0.081–0.772)	0.016*	0.248 (0.075–0.818)	0.022*

*p < 0.05, ***p < 0.005

We further evaluated the correlation between IL17A expression levels and various clinicopathological factors of HNSCC patients. Wilcoxon rank-sum test uncovered that decreased expression of IL17A was significantly correlated with patients’ OS event (*p* = 0.002, Fig. 1E), lymph vascular invasion (*p* = 0.003, Fig. 1F) and T stage (T3&T4 *vs.* T2&T1, *p* = 0.002, Fig. 1G).

Logistic regression analysis revealed that expression of IL17A is correlated with poor clinicopathologic and prognostic characteristics (Table 2). And decreased IL17A expression levels in HNSCC are significantly correlated with T stage (T3&T4 *vs.* T2&T1, *p* = 0.003) and lymph vascular invasion (yes *vs.* no, *p* = 0.012). Together, the above data indicated that HNSCC patients with low levels of IL17A expression are more prone to have tumors that are more advanced in the T stage and lymph vascular invasion compared to those with low levels of IL17A expression. Low levels of IL17A may influence tumorigenesis and progression in HNSCC.

**Table 2:** Association between IL17A expression and clinicopathologic characteristics using logistic regression

Characteristics	Total (*N*)	Odds ratio in IL17A expression	*P* value
T stage (T3 and T4 vs. T1 and T2)	487	0.572 (0.393–0.830)	0.003***
Age (> 60 *vs.* <= 60)	501	1.241 (0.874–1.764)	0.228
Gender (male vs. female)	502	0.922 (0.620–1.369)	0.687
N stage (N1 and N2 and N3 vs. N0)	480	0.860 (0.601–1.231)	0.410
M stage (M1 vs. M0)	477	0.644 (0.084–3.923)	0.632
Clinical stage (III and IV vs. I and II)	488	0.715 (0.467–1.088)	0.119
Histologic grade (G3 and G4 vs. G1 and G2)	483	1.028 (0.681–1.553)	0.896
Lymph vascular invasion (yes vs. no)	341	0.558 (0.353–0.876)	0.012*
Lymph node neck dissection (yes vs. no)	499	0.725 (0.456–1.145)	0.170

*p < 0.05, ***p < 0.005

### Identification of differentially expressed genes and enrichment analysis

**Fig. 2:**
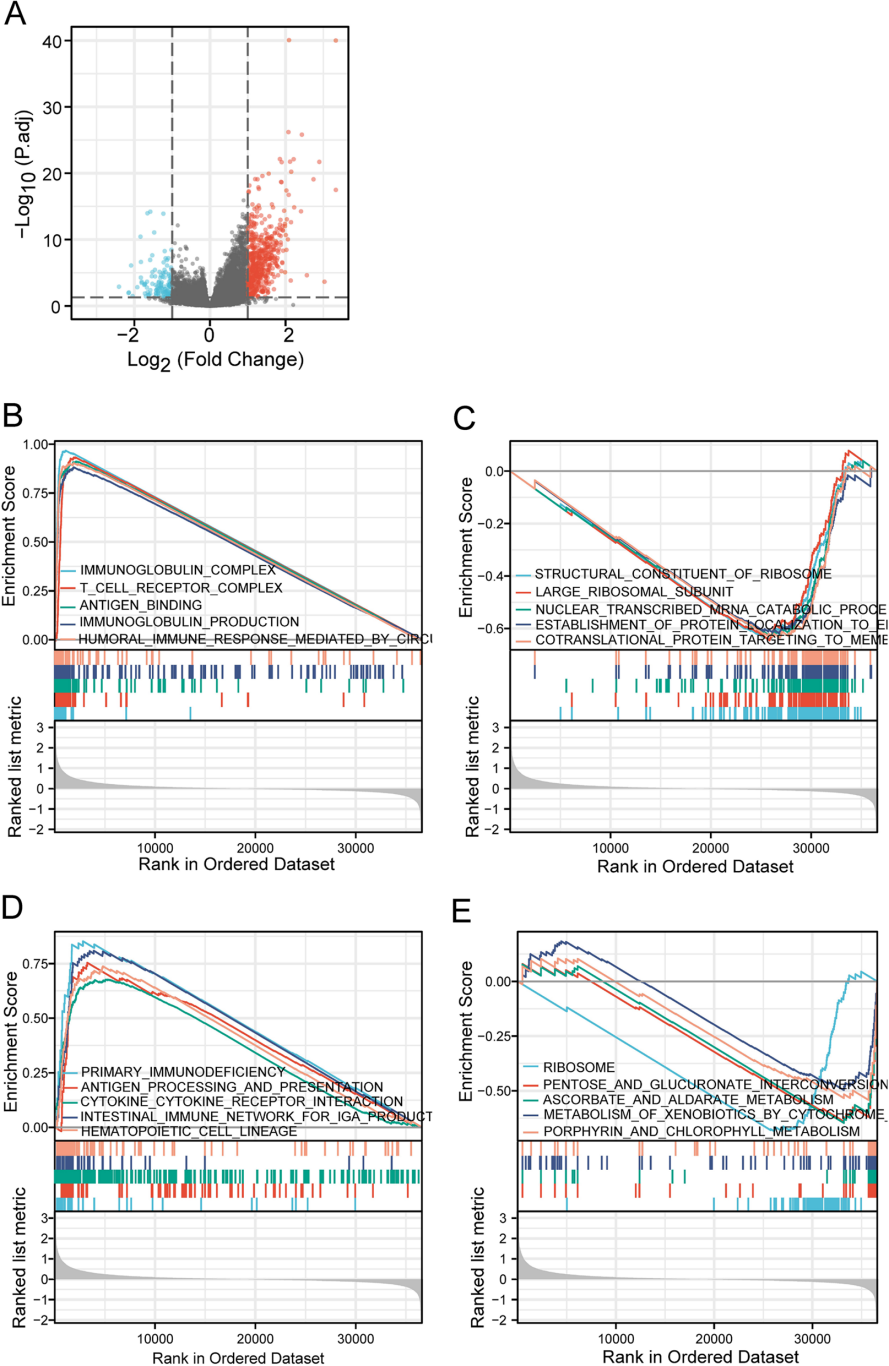
Volcano plot for differentially expressed genes between high and low expression of IL17A in HNSCC patients (**A**). GSEA revealed the top five positively correlated (**B**) and top five negatively correlated groups in GO term (**C**). GSEA revealed the top five positively correlated (**D**) and top five negatively correlated groups in the KEGG pathway (**E**)

A total of 661 genes (including 524 upregulated and 137 downregulated genes) were identified as DEGs between the high IL17A and low IL17A groups, as shown in the volcano plot (Fig. 2A). GSEA was performed to identify the potential biological function of IL17A. GSEA revealed significant differences in the enrichment of GO terms and KEGG pathways in samples between high and low levels of IL-17A. IL17A-related genes were assigned to 412 GO terms (|NES|) > 1, *p* < 0.05), including 399 positively (NES > 1) and 13 negatively (NES< − 1) correlations. We selected the most highly enriched signaling pathways based on their normalized enrichment score (NES) (Table 3). GO annotation uncovered five categories that were positively correlated with high levels of IL-17A: immunoglobulin complex, t cell receptor complex, antigen binding, immunoglobulin production and humoral immune response mediated by circulating immunoglobulin. GO analysis also revealed five negatively correlated categories: co-translational protein targeting to membrane, the establishment of protein localization to endoplasmic reticulum, nuclear-transcribed mRNA catabolic process nonsense-mediated decay, large ribosomal subunit, and structural constituent of ribosome (Fig. 2B, C, Supplementary Figure 2 and Supplementary Table 3).

**Table 3:** Signaling pathways most significantly correlated with IL17A expression based on their NES and FDR

	GO ID	NES	*p* value	FDR
Positive	Immunoglobulin complex	3.000	0.001	0.028
	T cell receptor complex	2.848	0.001	0.028
	Antigen binding	2.829	0.001	0.028
	Immunoglobulin production	2.799	0.001	0.028
	Humoral immune response mediated by circulating immunoglobulin	2.793	0.001	0.028
Negative	Co-translational protein targeting to membrane	− 2.337	0.006	0.073
	Establishment of protein localization to endoplasmic reticulum	− 2.346	0.007	0.078
	Nuclear transcribed mRNA catabolic process nonsense mediated decay	− 2.389	0.008	0.081
	Large ribosomal subunit	− 2.437	0.007	0.078
	Structural constituent of ribosome	− 2.545	0.010	0.097
	KEGG ID	NES	*p* value	FDR
Positive	Primary immunodeficiency	2.205	0.001	0.026
	Antigen processing and presentation	2.199	0.001	0.026
	Cytokine-cytokine receptor interaction	2.181	0.001	0.026
	Intestinal immune network for IgA production	2.180	0.001	0.026
	Hematopoietic cell lineage	2.171	0.001	0.026
Negative	Porphyrin and chlorophyll metabolism	− 1.691	0.011	0.081
	Metabolism of xenobiotics by cytochrome p450	− 1.712	0.014	0.096
	Ascorbate and aldarate metabolism	− 1.795	0.006	0.060
	Pentose and glucuronate interconversions	− 1.830	0.003	0.044
	Ribosome	− 2.458	0.006	0.060

KEGG pathway analysis showed the relevant genes were significantly enriched in 46 KEGG pathways, including 32 positively (NES > 1) and 4 negatively (NES< − 1) pathways. The five pathways that had the strongest positive correlation with IL17A expression: primary immunodeficiency, antigen processing and presentation, cytokine-cytokine receptor interaction, intestinal immune network for IgA production, and hematopoietic cell lineage. The five pathways with the strongest negative correlation were porphyrin and chlorophyll metabolism, metabolism of xenobiotics by cytochrome p450, ascorbate and aldarate metabolism, pentose and glucuronate interconversions, and ribosome (Fig. 2D, E, Supplementary Figure 2 and Supplementary Table 3). These results indicate that the pathways regulating humoral immune response, T cells response and cytokine signal, which are critically important in HNSCC patients, were strongly associated with IL17A expression.

### Correlation between IL17A expression and tumor-infiltration levels of immune cells in HNSCC

**Fig. 3:**
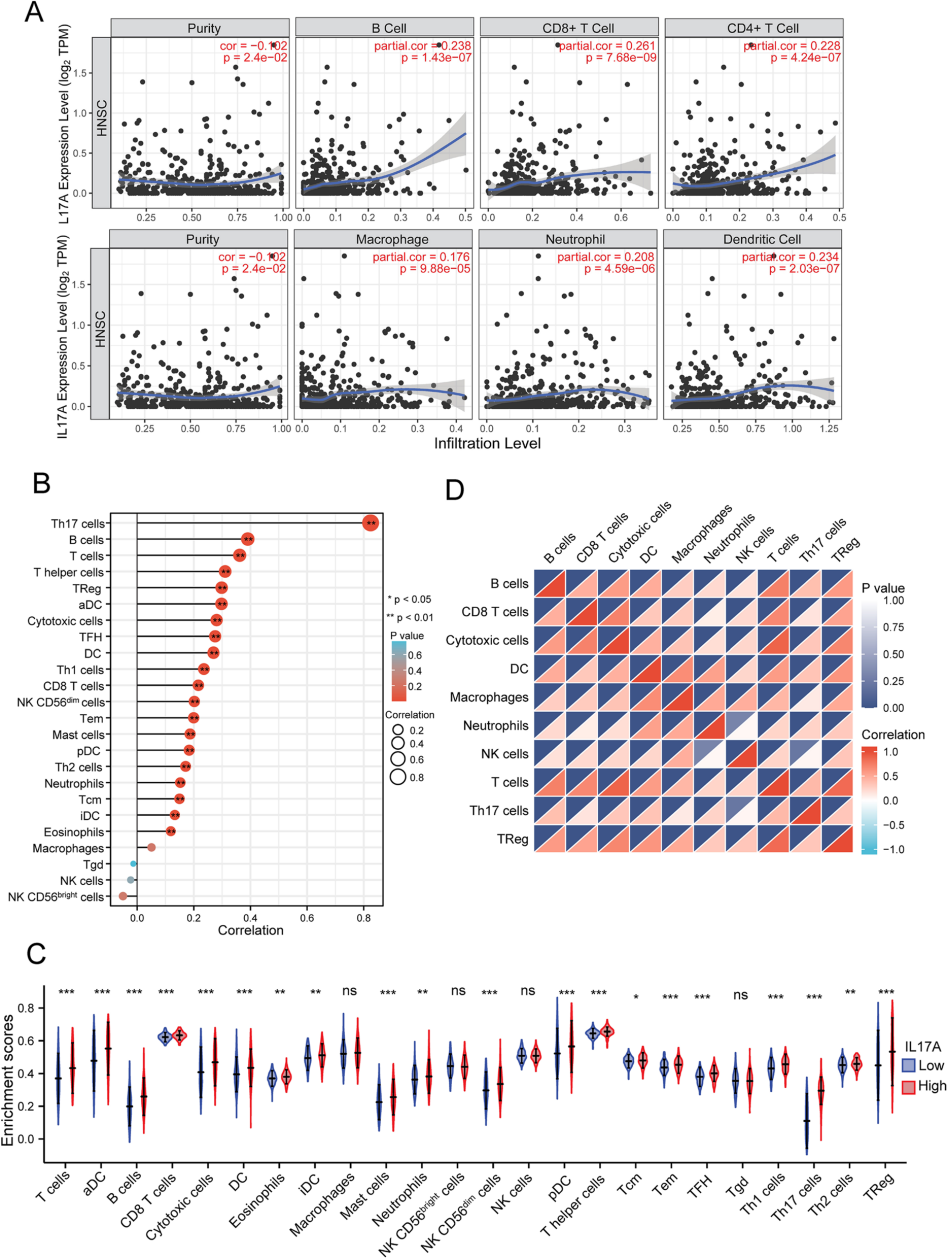
Scatterplots of correlations between IL17A expression and immune infiltration levels in HNSCC by TIMER (**A**). Correlations between IL17A expression and 24 subtypes of immune cells in TCGA HNSCC samples (**B**). The varied proportions of 24 subtypes of immune cells in high and low IL17A expression groups in HNSCC (**C**). Heatmap of immune infiltration cells in tumor samples (**D**). *p < 0.05, **p < 0.01, ***p < 0.005

Compelling evidence has demonstrated that TIICs are significantly associated with the prediction of overall survival rate and sentinel lymph node status. Therefore, we investigated whether IL17A expression was related to the levels of TIICs in HNSCC by TIMER. As shown in Fig. 3A, IL17A expression showed a positive correlation with the levels of B cells (*p* < 0.001), CD8^+^ T cells (*p* < 0.001) and CD4^+^ T cells (*p* < 0.001). The result was validated in the TCGA database, expression of IL17A was a positive correlation with the levels of B cells, T cells, and Th cells among 24 immune infiltration cell subtypes (*R*. > 0.3, *p* < 0.01 by Spearman, Fig. 3B, Table 4).

**Table 4:** Correlation analysis between IL17A and immune cells

Cell subset	Pearson		Spearman	
	*R.*	*P.*	*R.*	*P.*
Th17 cells	0.505	0.000	0.824	0.000
B cells	0.337	0.000	0.391	0.000
T cells	0.295	0.000	0.362	0.000
T helper cells	0.277	0.000	0.311	0.000
TReg	0.253	0.000	0.298	0.000
aDC	0.228	0.000	0.298	0.000
Cytotoxic cells	0.232	0.000	0.281	0.000
TFH	0.226	0.000	0.276	0.000
DC	0.163	0.000	0.270	0.000
Th1 cells	0.160	0.000	0.236	0.000
CD8 T cells	0.166	0.000	0.216	0.000
NK CD56dim cells	0.171	0.000	0.202	0.000
Tem	0.146	0.001	0.200	0.000
Mast cells	0.078	0.081	0.187	0.000
pDC	0.134	0.003	0.184	0.000
Th2 cells	0.172	0.000	0.171	0.000
Neutrophils	0.032	0.470	0.152	0.001
Tcm	0.140	0.002	0.150	0.001
iDC	0.027	0.551	0.133	0.003
Eosinophils	0.024	0.598	0.119	0.008
Macrophages	− 0.010	0.825	0.051	0.256
Tgd	− 0.059	0.184	− 0.013	0.765
NK cells	0.029	0.515	− 0.023	0.611
NK CD56bright cells	− 0.042	0.352	− 0.050	0.265

These results indicate that IL17A plays an important role in immune infiltration in HNSCC. Moreover, we tried to determine whether the tumor immune microenvironment was different in HNSCC cancer patients with high IL17A levels compared to those with low levels. Next, we assessed differences of the 24 immune infiltrated cell subtypes levels in tumor between high and low IL17A expression groups (Fig. 3C). B cells, T cells (including CD8^+^ T, Cytotoxic cells, T helper cells, and Treg), DCs, neutrophils and master cells were affected by IL17A expression. The heatmap of infiltration immune cells in tumor samples was also imaged to reveal the correlation of different type of TIICs (Fig. 3D).

**Fig. 4:**
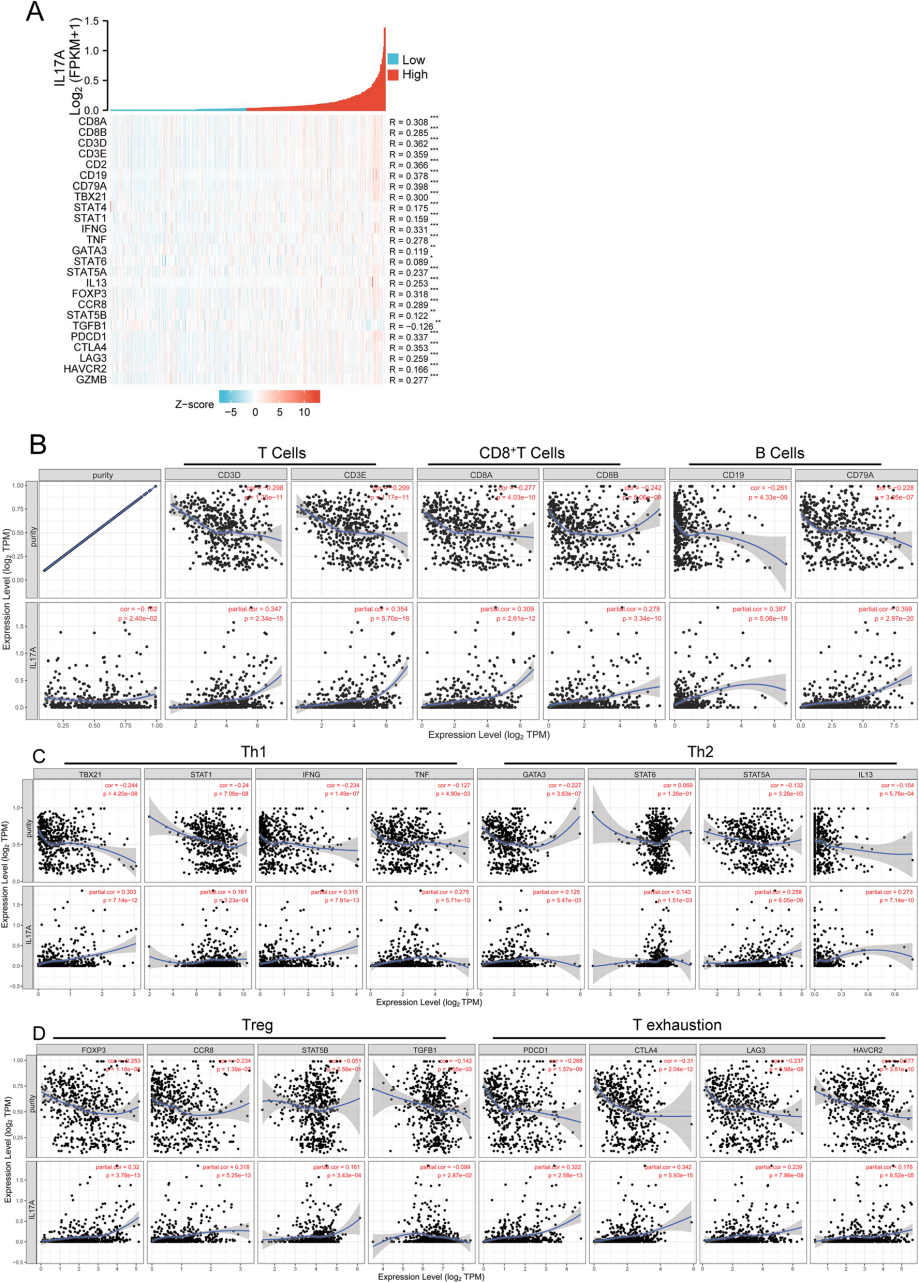
Correlation heat map between IL17A expression and immune marker genes of different immune cells (**A**). Scatterplots of correlations between IL17A expression and gene markers of CD8^+^ T cells, T cells, and B cells (**B**), Th1, Th2 (**C**), Treg and T exhaustion (**D**) in HNSCC. *p < 0.05, **p < 0.01, ***p < 0.005

Next, we validated the correlations between IL17A expression and immune marker genes of different immune cells, including CD8^+^T cells, T cells (general), B cells, monocytes, TAMs, M1 and M2 macrophages, neutrophils, and DCs in HNSCC by the TIMER. After the correlations were adjusted for purity, the results indicated that IL17A was significantly associated with immune markers of B cells and different T cells including exhaustion T cells in HNSCC (Table 5). Furthermore, we validated the relationship between IL17A expression and the markers of B cells and different T cells in HNSCC (Fig. 4 and Supplementary Table 4).

**Table 5:** Correlation analysis between IL17A and relate markers of immune cells

Cell subtype	Gene markers	None		Purity	
		Cor.	*P.*	Cor.	*P.*
CD8^+^ T cell	CD8A	0.323	0.000	0.309	0.000
	CD8B	0.293	0.000	0.278	0.000
T cell	CD3D	0.360	0.000	0.347	0.000
	CD3E	0.366	0.000	0.354	0.000
	CD2	0.372	0.000	0.359	0.000
B cell	CD19	0.398	0.000	0.387	0.000
	CD79A	0.410	0.000	0.399	0.000
Monocyte	CD86	0.192	0.000	0.171	0.000
	CSF1R	0.224	0.000	0.204	0.000
TAM	CCL2	0.231	0.000	0.213	0.000
	CD68	0.098	0.029	0.082	0.068
	IL10	0.297	0.000	0.281	0.000
M1	NOS2	0.273	0.000	0.283	0.000
	IRF5	0.120	0.008	0.120	0.008
	PTGS2	0.114	0.012	0.125	0.006
M2	CD163	0.145	0.001	0.122	0.007
	VSIG4	0.059	0.194	0.034	0.455
	MS4A4A	0.127	0.005	0.103	0.022
Neutrophils	CEACAM8	0.093	0.040	0.097	0.032
	ITGAM	0.179	0.000	0.167	0.000
	CCR7	0.381	0.000	0.370	0.000
	KIR2DL1	0.123	0.006	0.114	0.011
	KIR2DL3	0.137	0.002	0.125	0.006
	KIR2DL4	0.217	0.000	0.203	0.000
	KIR3DL1	0.217	0.000	0.205	0.000
	KIR3DL2	0.222	0.000	0.211	0.000
	KIR2DS4	0.082	0.069	0.068	0.132
Dendritic cell	HLA-DPB1	0.270	0.000	0.253	0.000
	HLA-DQB1	0.238	0.000	0.222	0.000
	HLA-DRA	0.275	0.000	0.258	0.000
	HLA-DPA1	0.263	0.000	0.246	0.000
	CD1C	0.243	0.000	0.225	0.000
	NRP1	0.025	0.576	0.004	0.924
	ITGAX	0.208	0.000	0.188	0.000
Th1	TBX21	0.317	0.000	0.303	0.000
	STAT4	0.188	0.000	0.168	0.000
	STAT1	0.180	0.000	0.161	0.000
	IFN-γ (IFNG)	0.329	0.000	0.315	0.000
	TNF-α (TNF)	0.284	0.000	0.275	0.000
Th2	GATA3	0.144	0.001	0.125	0.005
	STAT6	0.135	0.003	0.143	0.002
	STAT5A	0.268	0.000	0.258	0.000
	IL13	0.284	0.000	0.273	0.000
Tfh	BCL6	0.083	0.066	0.103	0.023
	IL21	0.355	0.000	0.344	0.000
Th17	STAT3	0.285	0.000	0.285	0.000
	IL17A	1.000	0.000	-1.000	0.000
Treg	FOXP3	0.333	0.000	0.320	0.000
	CCR8	0.331	0.000	0.318	0.000
	STAT5B	0.165	0.000	0.161	0.000
	TGF-β (TGFB1)	− 0.083	0.067	− 0.099	0.029
T cell exhaustion	PD-1 (PDCD1)	0.336	0.000	0.322	0.000
	CTLA4	0.355	0.000	0.342	0.000
	LAG3	0.255	0.000	0.239	0.000
	HAVCR2	0.197	0.000	0.176	0.000
	GZMB	0.281	0.000	0.265	0.000

### Correlation between IL17A expression and correlated genes top 20

**Fig. 5:**
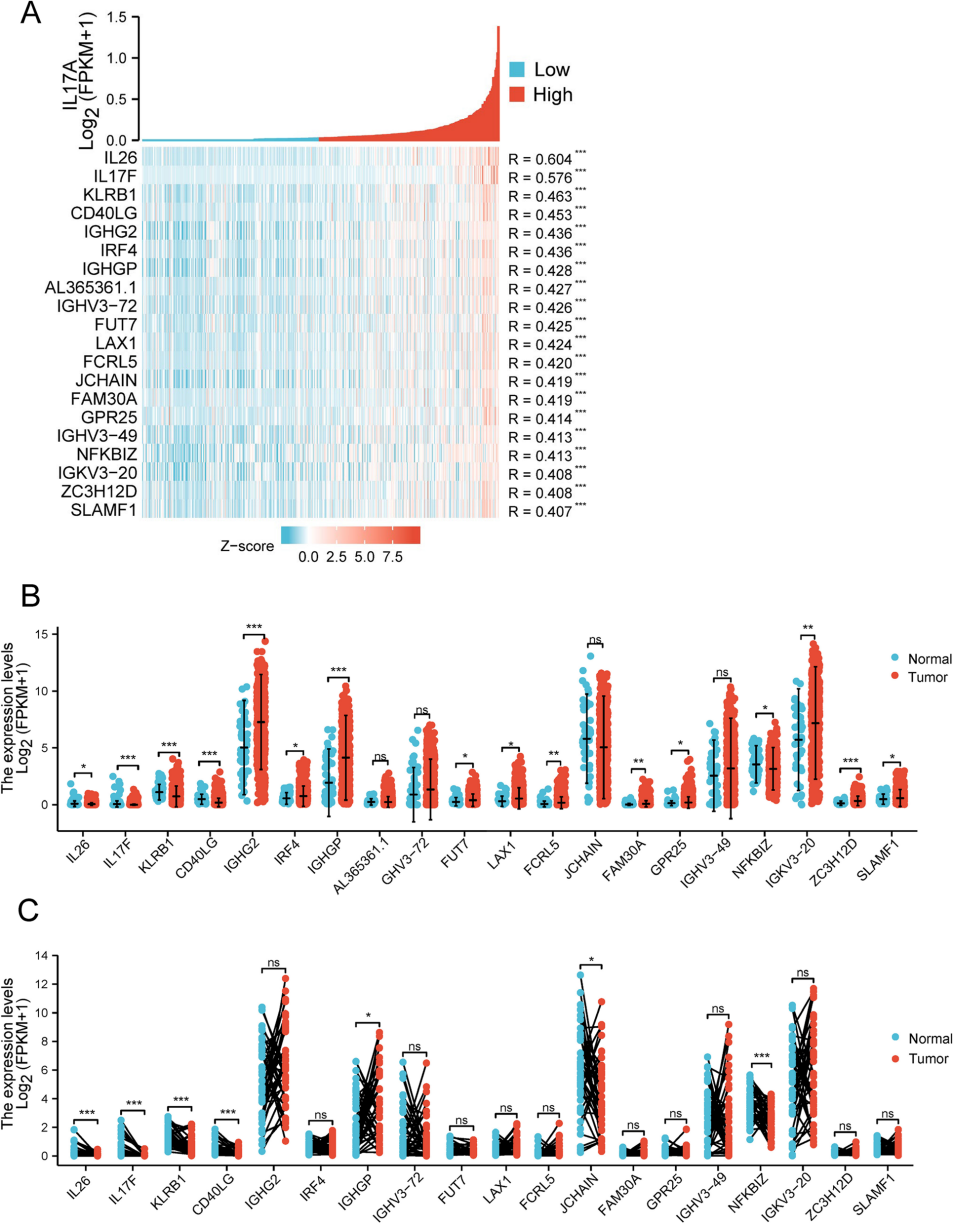
Correlation heat map between IL17A expression and correlated genes top 20 (**A**). The expression of IL17A correlated 20 genes between normal and HNSCC tumor tissues by unpaired sample analysis (**B**) and paired sample analysis (**C**). *p < 0.05, **p < 0.01, ***p < 0.005

**Fig. 6:**
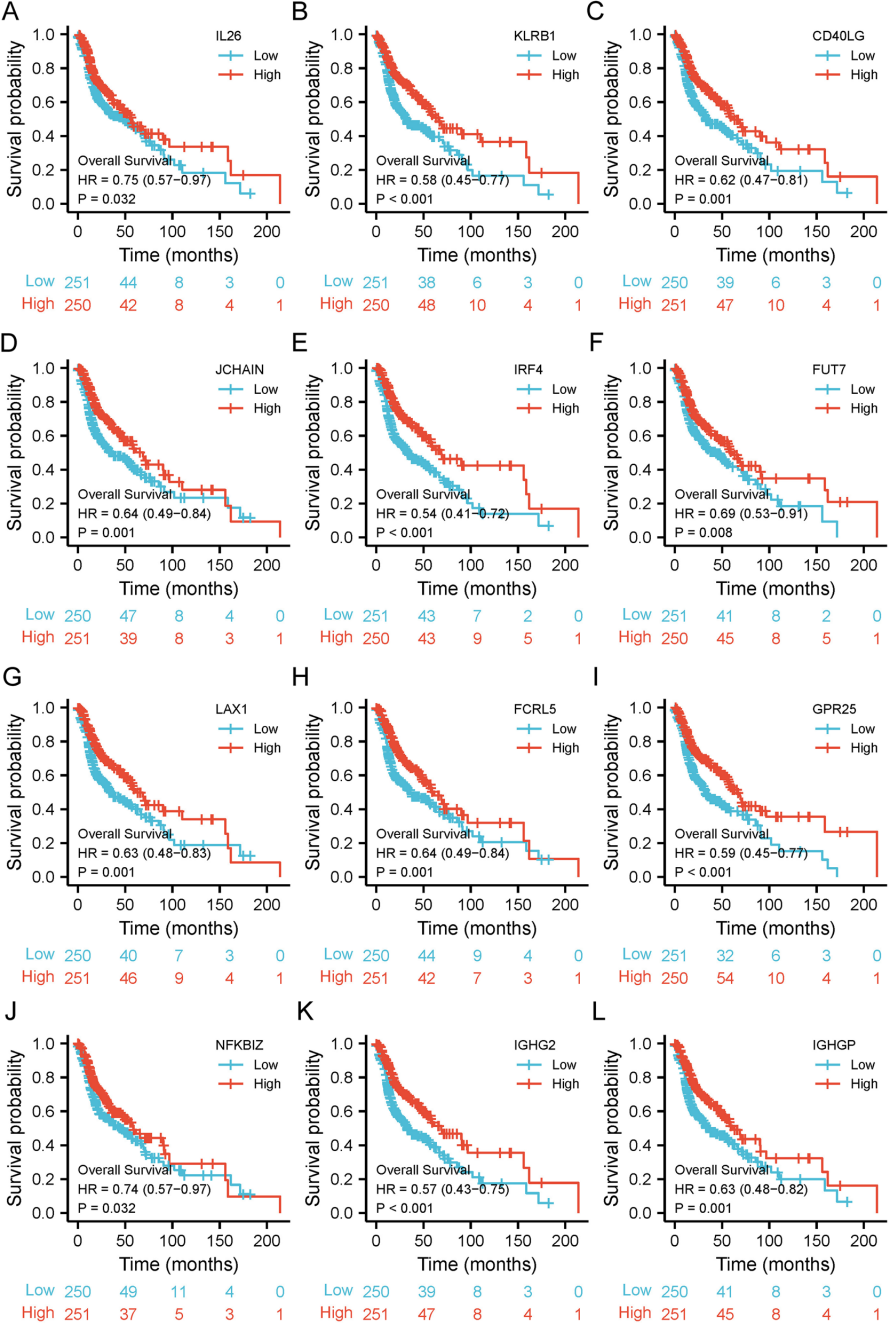
Kaplan–Meier survival curves (overall survival) comparing the high and low expression of the IL17A correlated 12 genes in HNSCC patients (**A**–**L**)

The correlation heat map showed the top 20 genes correlated with IL17A expression in HNSCC patients (*R* > 0.3, *p* < 0.001 by Spearman, Fig. 5A and Supplementary Table 5). The expression profiles of the top 20 genes correlated with IL17A in HNSCC and corresponding normal controls were investigated in the TCGA database (Fig. 5B unpaired samples and 5C paired samples). In unpaired analysis, we found that genes expression significantly lower in the tumor group were IL26, IL17F, KLRB1, CD40LG, genes expression significantly higher in the tumor group were IGHG2, IRF4, IGHGP, FUT7, LAX1 FCRL5, FAM30A, FPR25, NFKBIZ, IGKV3-20, ZC3H12D, and SLAMF1, compared with the normal group (Fig. 5B, and Supplementary Table 6). While, the paired analysis showed that expression of IL26, IL17F, KLRB1, CD40LG, JCHAIN, and NFKBIZ were significantly lower in the tumor group, compared with normal tissues (Fig. 5C, and Supplementary Table 6). Finally, we investigated the correlations between the IL17A correlated gene expression and prognosis in HNSCC in the TCGA database (Fig. 6 and Supplementary Figure 3).

## Discussion

IL-17A often referred to as IL-17, is a member of the IL-17 cytokine family that plays a key role in host immunity and protects the body from various microbial pathogens as well as tissue inflammation. IL-17A producing cells including Th17 cells (IL-17A producing CD4^+^ T helper cells) are involved in human cancers and autoimmune diseases [21, 22]. HNSCC is one of the most frequently occurring type of cancer. In the present study, we integrated IL17A expression and prognostic values in HNSCC using TCGA, GEPIA, TIMER databases and R. IL17A expression was decreased in tumor tissues compared with that in paired normal samples. Analysis of survival data from the TCGA database revealed that decreased IL17A expression is correlated with poor prognosis and IL17A expression is an independent factor for prognosis in HNSCC patients. HNSC patients with low IL17A expression are more likely to present a more advanced stage and lymphovascular invasion than those with high IL17A expression. The GSEA was performed to uncover the potential biological function of IL17A in HNSCC. GO term and KEGG pathway analysis revealed high levels of IL17A correlated with the humoral immune response, T cells response, and cytokine signal. Together with clinical correlation analysis, our data suggested that IL17A may play a tumor repression role in HNSCC patients.

The IL-17A response functions as a double-edged sword were suggested in cancers [23, 24]. Recent studies suggested a pathogenic role of IL-17A in cancer. For example, the IL17 and PI3K-Akt signaling pathway are enriched in oral cancer [25]. The canonical IL-23/IL-17A pathway is activated in the tracheal mucosa of idiopathic subglottic stenosis patients [26]. IL-17-producing γδ T cells and neutrophils conspire to promote breast cancer metastasis [27]. IL-17 and TNF-α up-regulate PD-L1 expression in human prostate and colon cancer [28]. Th17-IL-17 pathway plays a key role in prostate cancer progression, targeting IL17 pathway could prevent micro-invasive prostate cancer in a mouse model [29], and blocking IL-17A enhances tumor response to anti-PD-1 immunotherapy in microsatellite stable colorectal cancer [30]. While the other studies revealed the anti-tumor effect of IL17A. the IL17A deficient mice were more susceptible to developing lung melanoma, and Th17 cells promote the activation of tumor-specific CD8^+^ T cells [31]. Also, Th17 cells may contribute to protective tumor immunity by inducing Th1 chemokines and recruiting effector cells to the tumor microenvironment. Inhibition of Th17 cells represents a novel immune evasion mechanism [32]. HNSCC includes tumors with different genetic backgrounds and is characterized by highly clinical heterogeneity [33]. And the risk factors of HNSCC include smokeless tobacco and smoking, environmental pollutants exposure, human papillomavirus (HPV), and Epstein-Barr virus (EBV) infection [34]. Therefore, the potential biological function of IL17A may have two sides in individual cancer types of HNSCC. Single-cell sequencing and its applications may be a more efficient strategy to further uncover the prognostic biomarker in HNSCC [35].

The correlation between IL17A expression and diverse immune infiltration levels in HNSCC was analyzed in this study. Our data revealed that the expression of IL17A was moderately correlated with infiltration levels of B cells and T cells. Increased expression of IL17A was positively correlated with the expression of T cell markers, including total T cell markers CD3D, CD3E and CD2, CD8^+^T cell marker CD8A. In addition, increased expression of IL17A was also positively correlated with B cell markers CD19 and CD79A. The correlation between IL17A expression and immune cell marker indicated a key role for IL17A in regulating tumor immunology in HNSCC. The Treg markers FoxP3 and CCR8, the T exhaustion markers PDCD1 and CTLA4 [36–38] were correlated with IL17A expression. These correlations suggest a potential mechanism by which IL17A regulates T cells and B cell functions in HNSCC.

The most correlated with IL17A expression in HNSCC patients was IL26 (*R* = 0.604, *p* < 0.001). IL-26 is a member of the IL-10 cytokine family and plays a key regulatory role in multiple chronic inflammations and autoimmune diseases [39, 40]. Recently, a study showed that serum IL-26 levels were closely related to gastric cancer and its clinicopathological stages [41]. Our data showed that the expression of IL26 was significantly decreased in tumor tissue compared with corresponding normal control, and IL26 expression was significantly correlated with the prognosis of HNSCC. The second correlated with IL17A expression in HNSCC patients was IL17F (*R* = 0.576, *p* < 0.001). IL-17F, a member of the IL-17 cytokine family closest relative to IL-17A, signal through the same receptor complex (IL-17R) composed of the subunits IL-17RA and IL-17RC, may play a key role as well as IL-17A in HNSCC [23, 24]. Thus, the IL26 may be another important biomarker for elucidating how changes in cytokinesis and the immune environment promote the development of HNSCC.

Admittedly, there were several limitations in this study. First, we have exclusively used the public databases to analyze the correlation between IL17A expression and HNSCC occurrence and development. The results obtained need to be verified with more in vivo/vitro studies. Second, the expression of IL17A and correlated genes are analyzed based on the RNA-Seq database. The proteomic biomarkers, such as Pillai et al. recently reviewed in oral squamous cell cancer (OSCC), are unable to reflected in our current study [42]. Finally, the HNSCC includes tumors with different biologic backgrounds. Our study did not classify the epidemiologic backgrounds of HNSCC, and there may be some one-sidedness in the results.

## Conclusions

IL-17A plays a key role in HNSCC, involved in the regulation of immune cell infiltration, B cell and T cells responses. The levels of IL17A are important values for the determination of the occurrence and development of the HNSCC. The IL17A and correlated genes may be potential immunotherapeutic targets for HNSCC.

## Supplementary Information

**Additional file 1: Supplementary Table 1.** Clinical characteristics of HNSC patients. **Supplementary Table 2.** Correlation between overall survival and multivariable characteristics in TCGA patients. **Supplementary Table 3.** KEGG and GO term correlated with IL17A expression (Top 5). **Supplementary Table 4.** Correlation analysis between IL17A and relate genes markers of immune cells in HNSC. A TCGA. B GEPIA. **Supplementary Table 5.** Correlation analysis between IL17A and relate genes top 20. **Supplementary Table 6.** The genes expression between normal and tumor tissues. A Unpaired samples. Tumor=502, Normal=44. B Paired samples. N=44.

**Additional file 2: Figure 1. **The expression of IL17A between normal and HNSC tumor tissues(A). The correlations between the levels of IL17A expression and overall survival in HNSCC patients were verified in GEPIA (B) and UCSC Xena (C) online database. **Figure 2.** Pathway enrichment results for differentially expressed genes between high and low expression of IL17A in HNSC patients from KEGG and GO analysis (A, B). **Figure 3.** Kaplan Meier survival curves (disease specific survival) comparing the high and low expression of the IL17A correlated 12 genes in HNSC patients (A-L).

## Data Availability

The datasets used and/or analyzed during the current study are available from the corresponding author on reasonable request.

## Abbreviations

HNSCC: Head and neck squamous cell carcinoma
GEPIA: Gene Expression Profiling Interactive Analysis
GO: Gene Ontology
GSEA: Gene Set Enrichment Analysis
HNSCC: Head and neck squamous cell carcinoma
IL-17A: Interleukin-17A
KEGG: Kyoto Encyclopedia of Genes and Genomes
K-M survival: Kaplan-Meier survival
NES: Normalized enrichment score
TCGA: The Cancer Genome Atlas
TIIC: Tumor-infiltrating immune cells
TIMER: Tumor immune estimation resource
HPV: Human papillomavirus
EBV: Epstein Barr Virus
OSCC: Oral squamous cell cancer
