# Supplementary material for: Prognostic biomarker IL17A correlated with immune infiltrates in head and neck cancer
Source: World J Surg Oncol. 2022 Jul 28;20:243. doi: 10.1186/s12957-022-02703-1 (PMC9330648; doi:10.1186/s12957-022-02703-1)
Supplement: Supplementary file 1 — Additional file 1: Supplementary Table 1. Clinical characteristics of HNSC patients. Supplementary Table 2. Correlation between overall survival and multivariable characteristics in TCGA patients. Supplementary Table 3. KEGG and GO term correlated with IL17A expression (Top 5). Supplementary Table 4. Correlation analysis between IL17A and relate genes markers of immune cells in HNSC. A TCGA. B GEPIA. Supplementary Table 5. Correlation analysis between IL17A and relate genes top 20. Supplementary Table 6. The genes expression between normal and tumor tissues. A Unpaired samples. Tumor=502, Normal=44. B Paired samples. N=44. [file 12957_2022_2703_MOESM1_ESM.docx]

**Supplementary Table 1.** Clinical characteristics of HNSC patients

| Characteristic | Low expression of IL17A | High expression of IL17A | *p* |
| --- | --- | --- | --- |
| n | 251 | 251 |  |
| T stage, n (%) |  |  | **0.004***** |
| T1 | 14 (2.9%) | 19 (3.9%) |  |
| T2 | 58 (11.9%) | 86 (17.7%) |  |
| T3 | 62 (12.7%) | 69 (14.2%) |  |
| T4 | 107 (22%) | 72 (14.8%) |  |
| N stage, n (%) |  |  | 0.281 |
| N0 | 113 (23.5%) | 126 (26.2%) |  |
| N1 | 47 (9.8%) | 33 (6.9%) |  |
| N2 | 72 (15%) | 82 (17.1%) |  |
| N3 | 4 (0.8%) | 3 (0.6%) |  |
| M stage, n (%) |  |  | 0.682 |
| M0 | 232 (48.6%) | 240 (50.3%) |  |
| M1 | 3 (0.6%) | 2 (0.4%) |  |
| Clinical stage, n (%) |  |  | 0.269 |
| Stage I | 6 (1.2%) | 13 (2.7%) |  |
| Stage II | 43 (8.8%) | 52 (10.7%) |  |
| Stage III | 50 (10.2%) | 52 (10.7%) |  |
| Stage IV | 142 (29.1%) | 130 (26.6%) |  |
| Radiation therapy, n (%) |  |  | 0.798 |
| No | 74 (16.8%) | 80 (18.1%) |  |
| Yes | 143 (32.4%) | 144 (32.7%) |  |
| Primary therapy outcome, n (%) |  |  | 0.180 |
| PD | 23 (5.5%) | 18 (4.3%) |  |
| SD | 2 (0.5%) | 4 (1%) |  |
| PR | 5 (1.2%) | 1 (0.2%) |  |
| CR | 170 (40.7%) | 195 (46.7%) |  |
| Gender, n (%) |  |  | 0.762 |
| Female | 65 (12.9%) | 69 (13.7%) |  |
| Male | 186 (37.1%) | 182 (36.3%) |  |
| Race, n (%) |  |  | 0.763 |
| Asian | 4 (0.8%) | 6 (1.2%) |  |
| Black or African American | 25 (5.2%) | 22 (4.5%) |  |
| White | 215 (44.3%) | 213 (43.9%) |  |
| Age, n (%) |  |  | 0.264 |
| <=60 | 129 (25.7%) | 116 (23.2%) |  |
| >60 | 121 (24.2%) | 135 (26.9%) |  |
| Histologic grade, n (%) |  |  | 0.838 |
| G1 | 28 (5.8%) | 34 (7%) |  |
| G2 | 154 (31.9%) | 146 (30.2%) |  |
| G3 | 59 (12.2%) | 60 (12.4%) |  |
| G4 | 1 (0.2%) | 1 (0.2%) |  |
| Anatomic neoplasm subdivision, n (%) |  |  | 0.195 |
| Alveolar Ridge | 11 (2.2%) | 7 (1.4%) |  |
| Base of tongue | 10 (2%) | 13 (2.6%) |  |
| Buccal Mucosa | 16 (3.2%) | 6 (1.2%) |  |
| Floor of mouth | 32 (6.4%) | 29 (5.8%) |  |
| Hard Palate | 2 (0.4%) | 5 (1%) |  |
| Hypopharynx | 5 (1%) | 5 (1%) |  |
| Larynx | 56 (11.2%) | 55 (11%) |  |
| Lip | 1 (0.2%) | 2 (0.4%) |  |
| Oral Cavity | 41 (8.2%) | 31 (6.2%) |  |
| Oral Tongue | 57 (11.4%) | 69 (13.7%) |  |
| Oropharynx | 6 (1.2%) | 3 (0.6%) |  |
| Tonsil | 14 (2.8%) | 26 (5.2%) |  |
| Lymphovascular invasion, n (%) |  |  | **0.016*** |
| No | 107 (31.4%) | 112 (32.8%) |  |
| Yes | 77 (22.6%) | 45 (13.2%) |  |
| Lymphnode neck dissection, n (%) |  |  | 0.208 |
| No | 39 (7.8%) | 51 (10.2%) |  |
| Yes | 210 (42.1%) | 199 (39.9%) |  |
| Age, median (IQR) | 60 (53, 67.75) | 61 (53.5, 69.5) | 0.463 |

*p<0.05, ***p<0.005.

**Supplementary Table 2.** Correlation between overall survival and multivariable characteristics in TCGA patients

| Characteristics | Total (N) | Univariate analysis | |  | Multivariate analysis | |
| --- | --- | --- | --- | --- | --- | --- |
|  |  | Hazard ratio (95% CI) | P value |  | Hazard ratio (95% CI) | P value |
| Age | 501 |  |  |  |  |  |
| <=60 | 245 | Reference |  |  |  |  |
| >60 | 256 | 1.252 (0.956-1.639) | 0.102 |  | 1.294 (0.971-1.725) | 0.079 |
| Gender | 501 |  |  |  |  |  |
| Female | 134 | Reference |  |  |  |  |
| Male | 367 | 0.764 (0.574-1.018) | 0.066 |  | 0.831 (0.613-1.128) | 0.235 |
| Clinical stage | 487 |  |  |  |  |  |
| Stage I&Stage II | 113 | Reference |  |  |  |  |
| Stage III&Stage IV | 374 | 1.217 (0.878-1.688) | 0.238 |  | 0.748 (0.411-1.362) | 0.342 |
| T stage | 486 |  |  |  |  |  |
| T1&T2 | 176 | Reference |  |  |  |  |
| T3&T4 | 310 | 1.245 (0.932-1.661) | 0.137 |  | 1.459 (0.879-2.424) | 0.144 |
| M stage | 476 |  |  |  |  |  |
| M0 | 471 | Reference |  |  |  |  |
| M1 | 5 | 4.745 (1.748-12.883) | 0.002*** |  | 4.853 (1.730-13.610) | **0.003***** |
| N stage | 479 |  |  |  |  |  |
| N0&N1 | 318 | Reference |  |  |  |  |
| N2&N3 | 161 | 1.384 (1.040-1.842) | 0.026* |  | 1.502 (1.090-2.068) | **0.013*** |
| IL17A | 501 | 0.250 (0.081-0.772) | 0.016* |  | 0.248 (0.075-0.818) | **0.022*** |

*p<0.05, ***p<0.005.

**Supplementary Table 3.** KEGG and GO term correlated with IL17A expression (Top 5)

| Ontology | ID | Description | GeneRatio | pvalue | p.adjust | qvalue |
| --- | --- | --- | --- | --- | --- | --- |
| BP | GO:0006958 | complement activation, classical pathway | 83/282 | 0.000 | 0.000 | 0.000 |
| BP | GO:0002455 | humoral immune response mediated by circulating immunoglobulin | 84/282 | 0.000 | 0.000 | 0.000 |
| BP | GO:0006956 | complement activation | 84/282 | 0.000 | 0.000 | 0.000 |
| BP | GO:0016064 | immunoglobulin mediated immune response | 88/282 | 0.000 | 0.000 | 0.000 |
| BP | GO:0019724 | B cell mediated immunity | 88/282 | 0.000 | 0.000 | 0.000 |
| CC | GO:0019814 | immunoglobulin complex | 113/285 | 0.000 | 0.000 | 0.000 |
| CC | GO:0042571 | immunoglobulin complex, circulating | 50/285 | 0.000 | 0.000 | 0.000 |
| CC | GO:0009897 | external side of plasma membrane | 59/285 | 0.000 | 0.000 | 0.000 |
| CC | GO:0042101 | T cell receptor complex | 37/285 | 0.000 | 0.000 | 0.000 |
| CC | GO:0098802 | plasma membrane receptor complex | 39/285 | 0.000 | 0.000 | 0.000 |
| MF | GO:0003823 | antigen binding | 83/209 | 0.000 | 0.000 | 0.000 |
| MF | GO:0034987 | immunoglobulin receptor binding | 49/209 | 0.000 | 0.000 | 0.000 |
| MF | GO:0048018 | receptor ligand activity | 18/209 | 0.000 | 0.002 | 0.002 |
| MF | GO:0016493 | C-C chemokine receptor activity | 4/209 | 0.000 | 0.010 | 0.009 |
| MF | GO:0019957 | C-C chemokine binding | 4/209 | 0.000 | 0.010 | 0.009 |
| KEGG | hsa04060 | Cytokine-cytokine receptor interaction | 16/73 | 0.000 | 0.000 | 0.000 |
| KEGG | hsa05340 | Primary immunodeficiency | 5/73 | 0.000 | 0.001 | 0.001 |
| KEGG | hsa04061 | Viral protein interaction with cytokine and cytokine receptor | 7/73 | 0.000 | 0.001 | 0.001 |
| KEGG | hsa05321 | Inflammatory bowel disease | 5/73 | 0.000 | 0.011 | 0.009 |
| KEGG | hsa04659 | Th17 cell differentiation | 6/73 | 0.000 | 0.012 | 0.010 |

**Supplementary Table 4.** Correlation analysis between IL17A and relate genes markers of immune cells in HNSC

A. TCGA

| Cell subtype | Gene markers | Pearson | |  | Spearman | | |  |
| --- | --- | --- | --- | --- | --- | --- | --- | --- |
|  |  | R. | P. |  | R. | | P. |  |
| CD8 + T cell | CD8A | 0.278 | 0.000 |  | 0.308 | | 0.000 |  |
|  | CD8B | 0.243 | 0.000 |  | 0.285 | | 0.000 |  |
| T cell | CD3D | 0.297 | 0.000 |  | 0.362 | | 0.000 |  |
|  | CD3E | 0.321 | 0.000 |  | 0.359 | | 0.000 |  |
|  | CD2 | 0.306 | 0.000 |  | 0.366 | | 0.000 |  |
| B cell | CD19 | 0.342 | 0.000 |  | 0.378 | | 0.000 |  |
|  | CD79A | 0.391 | 0.000 |  | 0.398 | | 0.000 |  |
| Th1 | TBX21 | 0.271 | 0.000 |  | 0.300 | | 0.000 |  |
|  | STAT4 | 0.088 | 0.049 |  | 0.175 | | 0.000 |  |
|  | STAT1 | 0.079 | 0.077 |  | 0.159 | | 0.000 |  |
|  | IFN-γ (IFNG) | 0.266 | 0.000 |  | 0.331 | | 0.000 |  |
|  | TNF-α (TNF) | 0.150 | 0.001 |  | 0.278 | | 0.000 |  |
| Th2 | GATA3 | 0.093 | 0.038 |  | 0.119 | | 0.008 |  |
|  | STAT6 | 0.056 | 0.207 |  | 0.089 | | 0.047 |  |
|  | STAT5A | 0.199 | 0.000 |  | | 0.237 | 0.000 | |
|  | IL13 | 0.231 | 0.000 |  | | 0.253 | 0.000 | |
| Treg | FOXP3 | 0.285 | 0.000 |  | | 0.318 | 0.000 | |
|  | CCR8 | 0.226 | 0.000 |  | | 0.289 | 0.000 | |
|  | STAT5B | 0.067 | 0.132 |  | | 0.122 | 0.006 | |
|  | TGF-β (TGFB1) | -0.156 | 0.000 |  | | -0.126 | 0.005 | |
| T cell exhaustion | PD-1 (PDCD1) | 0.327 | 0.000 |  | | 0.337 | 0.000 | |
|  | CTLA4 | 0.272 | 0.000 |  | | 0.353 | 0.000 | |
|  | LAG3 | 0.192 | 0.000 |  | | 0.259 | 0.000 | |
|  | HAVCR2 | 0.146 | 0.001 |  | | 0.166 | 0.000 | |
|  | GZMB | 0.213 | 0.000 |  | | 0.277 | 0.000 | |

B． GEPIA

| Cell subtype | Gene markers | Tumor | |  | Normal | | |  |
| --- | --- | --- | --- | --- | --- | --- | --- | --- |
|  |  | R. | P. |  | R. | | P. |  |
| CD8 + T cell | CD8A | 0.34 | 0.000 |  | 0.24 | | 0.010 |  |
|  | CD8B | 0.3 | 0.000 |  | 0.08 | | 0.580 |  |
| T cell | CD3D | 0.38 | 0.000 |  | 0.61 | | 0.000 |  |
|  | CD3E | 0.38 | 0.000 |  | 0.61 | | 0.000 |  |
|  | CD2 | 0.38 | 0.000 |  | 0.58 | | 0.000 |  |
| B cell | CD19 | 0.39 | 0.000 |  | 0.19 | | 0.210 |  |
|  | CD79A | 0.41 | 0.000 |  | 0.13 | | 0.390 |  |
| Th1 | TBX21 | 0.32 | 0.000 |  | 0.3 | | 0.048 |  |
|  | STAT4 | 0.19 | 0.000 |  | 0.51 | | 0.000 |  |
|  | STAT1 | 0.19 | 0.000 |  | 0.36 | | 0.016 |  |
|  | IFN-γ (IFNG) | 0.35 | 0.000 |  | 0.30 | | 0.045 |  |
|  | TNF-α (TNF) | 0.29 | 0.000 |  | 0.25 | | 0.100 |  |
| Th2 | GATA3 | 0.13 | 0.004 |  | 0.04 | | 0.780 |  |
|  | STAT6 | 0.13 | 0.004 |  | 0.37 | | 0.013 |  |
|  | STAT5A | 0.27 | 0.000 |  | | 0.16 | 0.290 | |
|  | IL13 | 0.30 | 0.000 |  | | 0.12 | 0.440 | |
| Treg | FOXP3 | 0.35 | 0.000 |  | | 0.49 | 0.000 | |
|  | CCR8 | 0.34 | 0.000 |  | | 0.34 | 0.023 | |
|  | STAT5B | 0.15 | 0.000 |  | | 0.17 | 0.280 | |
|  | TGF-β (TGFB1) | -0.13 | 0.004 |  | | 0.33 | 0.027 | |
| T cell exhaustion | PD-1 (PDCD1) | 0.36 | 0.000 |  | | 0.41 | 0.006 | |
|  | CTLA4 | 0.37 | 0.000 |  | | 0.69 | 0.000 | |
|  | LAG3 | 0.26 | 0.000 |  | | 0.40 | 0.008 | |
|  | HAVCR2 | 0.20 | 0.000 |  | | 0.20 | 0.200 | |
|  | GZMB | 0.28 | 0.000 |  | | 0.60 | 0.000 | |

**Supplementary Table 5.** Correlation analysis between IL17A and relate genes top 20

| Gene name | Gene biotype | Pearson | |  | Spearman | | |  |
| --- | --- | --- | --- | --- | --- | --- | --- | --- |
|  |  | R. | P. |  | R. | | P. |  |
| IL26 | Protein coding | 0.666 | 0.000 |  | 0.604 | | 0.000 |  |
| IL17F | Protein coding | 0.666 | 0.000 |  | 0.576 | | 0.000 |  |
| KLRB1 | Protein coding | 0.439 | 0.000 |  | 0.463 | | 0.000 |  |
| CD40LG | Protein coding | 0.385 | 0.000 |  | 0.453 | | 0.000 |  |
| IGHG2 | IG C gene | 0.359 | 0.000 |  | 0.436 | | 0.000 |  |
| IRF4 | Protein coding | 0.414 | 0.000 |  | 0.436 | | 0.000 |  |
| IGHGP | IG C pseudogene | 0.367 | 0.000 |  | 0.428 | | 0.000 |  |
| AL365361.1 | lncRNA | 0.396 | 0.000 |  | 0.427 | | 0.000 |  |
| IGHV3-72 | IG V gene | 0.368 | 0.000 |  | 0.426 | | 0.000 |  |
| FUT7 | Protein coding | 0.329 | 0.000 |  | 0.425 | | 0.000 |  |
| LAX1 | Protein coding | 0.418 | 0.000 |  | 0.424 | | 0.000 |  |
| FCRL5 | Protein coding | 0.402 | 0.000 |  | 0.420 | | 0.000 |  |
| JCHAIN | Protein coding | 0.354 | 0.000 |  | 0.419 | | 0.000 |  |
| FAM30A | lncRNA | 0.361 | 0.000 |  | 0.419 | | 0.000 |  |
| GPR25 | Protein coding | 0.427 | 0.000 |  | | 0.414 | 0.000 | |
| IGHV3-49 | IG V gene | 0.341 | 0.000 |  | | 0.413 | 0.000 | |
| NFKBIZ | Protein coding | 0.367 | 0.000 |  | | 0.413 | 0.000 | |
| IGKV3-20 | IG V gene | 0.364 | 0.000 |  | | 0.408 | 0.000 | |
| ZC3H12D | Protein coding | 0.383 | 0.000 |  | | 0.408 | 0.000 | |
| SLAMF1 | Protein coding | 0.400 | 0.000 |  | | 0.407 | 0.000 | |

**Supplementary Table 6.** The genes expression between normal and tumor tissues

1. unpaired samples

| Group | Changes(T-N) | 95% CI-L | 95% CI-H | p-value |
| --- | --- | --- | --- | --- |
| IL26 | -0.038 | -0.077 | -0.002 | 0.012 |
| IL17F | -0.042 | -0.098 | -0.019 | 0.000 |
| KLRB1 | -0.363 | -0.542 | -0.177 | 0.000 |
| CD40LG | -0.176 | -0.252 | -0.104 | 0.000 |
| IGHG2 | 2.005 | 1.100 | 2.904 | 0.000 |
| IRF4 | 0.153 | 0.004 | 0.321 | 0.044 |
| IGHGP | 1.669 | 0.925 | 2.425 | 0.000 |
| AL365361.1 | 0.022 | -0.042 | 0.098 | 0.538 |
| IGHV3-72 | 0.204 | -0.066 | 0.639 | 0.166 |
| FUT7 | 0.091 | 0.003 | 0.185 | 0.041 |
| LAX1 | 0.135 | 0.026 | 0.276 | 0.013 |
| FCRL5 | 0.069 | 0.020 | 0.137 | 0.001 |
| JCHAIN | -0.817 | -1.687 | 0.005 | 0.052 |
| FAM30A | 0.034 | 0.010 | 0.069 | 0.002 |
| GPR25 | 0.058 | 0.004 | 0.132 | 0.027 |
| IGHV3-49 | 0.539 | -0.035 | 1.210 | 0.068 |
| NFKBIZ | -0.426 | -0.779 | -0.079 | 0.016 |
| IGKV3-20 | 1.280 | 0.388 | 2.172 | 0.005 |
| ZC3H12D | 0.162 | 0.108 | 0.221 | 0.000 |
| SLAMF1 | 0.115 | 0.006 | 0.229 | 0.036 |

Tumor=502, Normal=44

1. paired samples

| Group | Changes(T-N) | 95% CI-L | 95% CI-H | p-value |
| --- | --- | --- | --- | --- |
| IL26 | -0.103 | -0.242 | -0.040 | 0.001 |
| IL17F | -0.208 | -0.439 | -0.100 | 0.000 |
| KLRB1 | -0.573 | -0.828 | -0.331 | 0.000 |
| CD40LG | -0.297 | -0.428 | -0.189 | 0.000 |
| IGHG2 | 1.172 | -0.119 | 2.689 | 0.065 |
| IRF4 | -0.026 | -0.211 | 0.205 | 0.872 |
| IGHGP | 1.093 | 0.056 | 2.256 | 0.044 |
| AL365361.1 | -0.180 | -0.920 | 0.391 | 0.530 |
| IGHV3-72 | -0.035 | -0.107 | 0.030 | 0.278 |
| FUT7 | -0.035 | -0.107 | 0.030 | 0.278 |
| LAX1 | 0.079 | -0.074 | 0.281 | 0.311 |
| FCRL5 | 0.078 | -0.024 | 0.224 | 0.209 |
| JCHAIN | -1.310 | -2.416 | -0.149 | 0.027 |
| FAM30A | 0.011 | -0.036 | 0.093 | 0.680 |
| GPR25 | 0.036 | -0.059 | 0.137 | 0.499 |
| IGHV3-49 | -0.116 | -1.094 | 0.936 | 0.812 |
| NFKBIZ | -0.893 | -1.331 | -0.444 | 0.000 |
| IGKV3-20 | 0.598 | -0.763 | -1.720 | 0.366 |
| ZC3H12D | 0.054 | 0 .000 | 0.137 | 0.052 |
| SLAMF1 | -0.008 | -0.156 | 0.124 | 0.900 |

N=44
