# Supplementary material for: Prognostic biomarker IL17A correlated with immune infiltrates in head and neck cancer
Source: World J Surg Oncol. 2022 Jul 28;20:243. doi: 10.1186/s12957-022-02703-1 (PMC9330648; doi:10.1186/s12957-022-02703-1)
Supplement: Supplementary file 2 — Additional file 2: Figure 1. The expression of IL17A between normal and HNSC tumor tissues(A). The correlations between the levels of IL17A expression and overall survival in HNSCC patients were verified in GEPIA (B) and UCSC Xena (C) online database. Figure 2. Pathway enrichment results for differentially expressed genes between high and low expression of IL17A in HNSC patients from KEGG and GO analysis (A, B). Figure 3. Kaplan Meier survival curves (disease specific survival) comparing the high and low expression of the IL17A correlated 12 genes in HNSC patients (A-L). [file 12957_2022_2703_MOESM2_ESM.pdf]

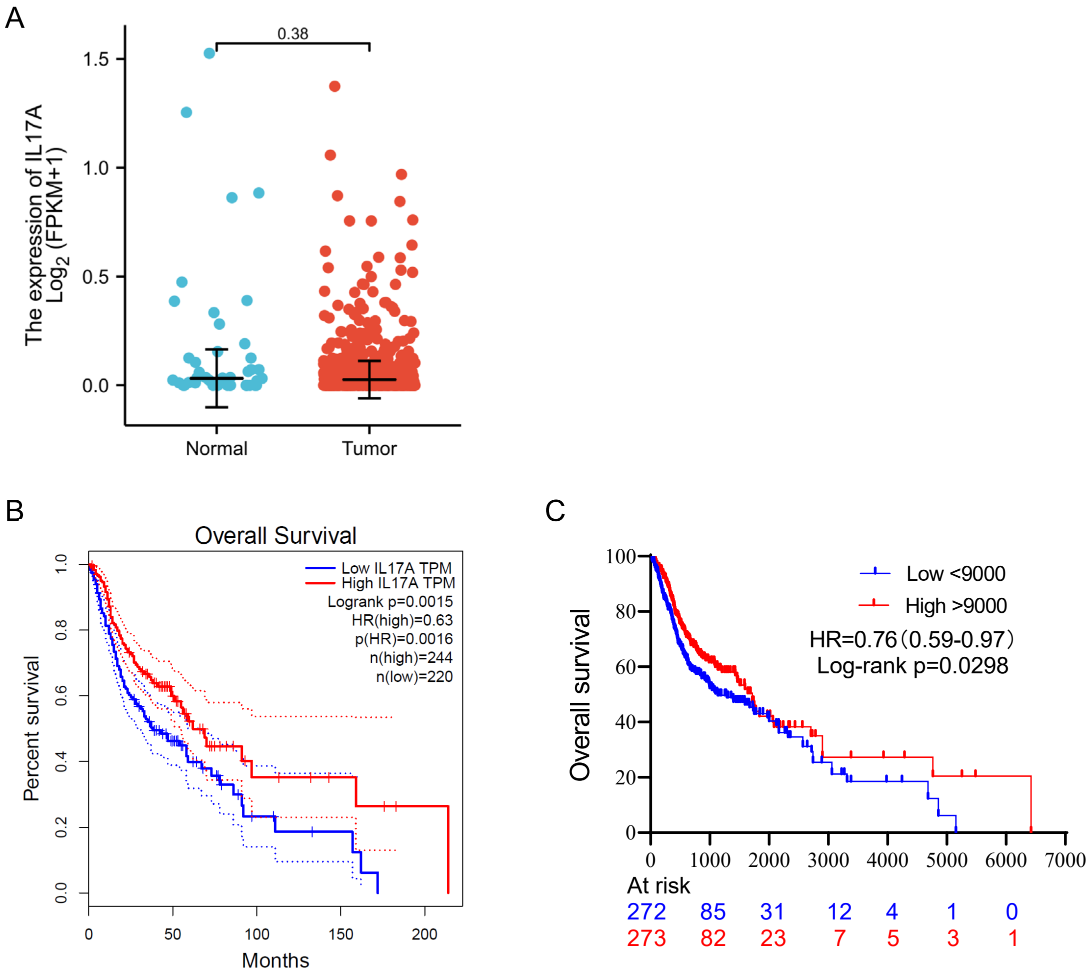

Figure 1. The expression of IL17A between normal and HNSC tumor tissues(A). The correlations between the levels of IL17A expression and overall survival in HNSCC patients were verified in GEPIA (B) and UCSC Xena (C) online database.

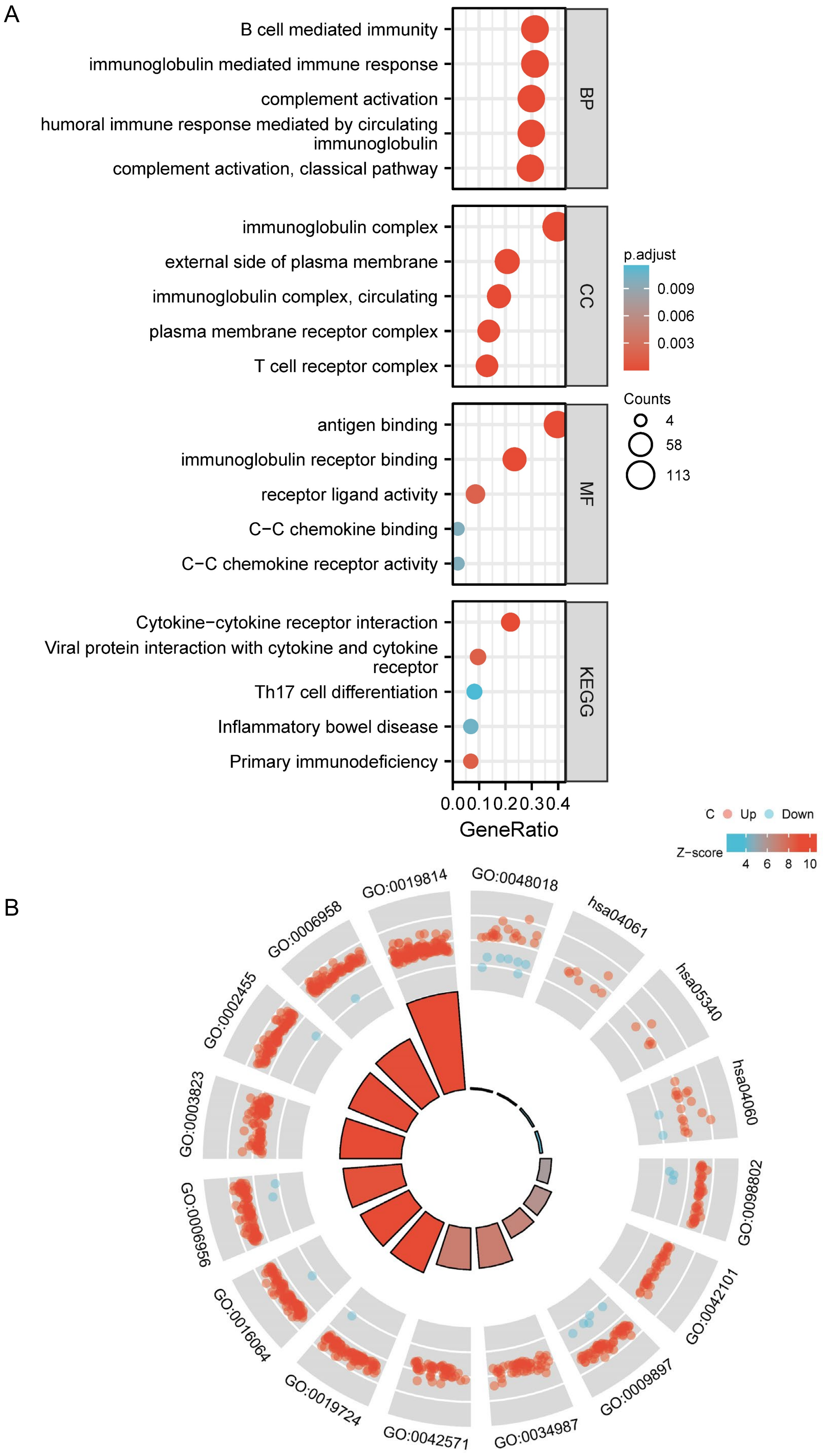

Figure 2. Pathway enrichment results for differentially expressed genes between high and low expression of IL17A in HNSC patients from KEGG and GO analysis (A, B).

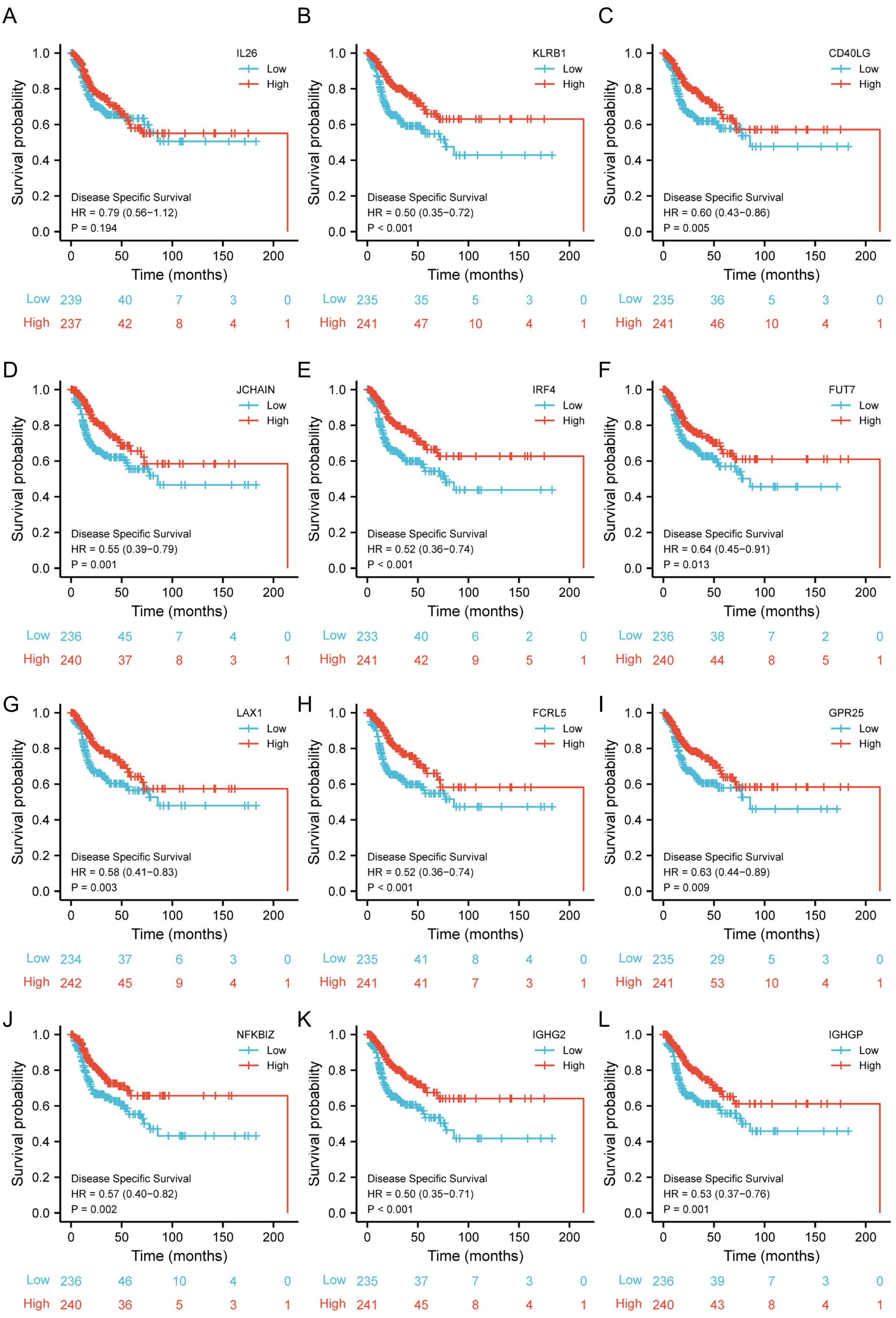

Figure 3. Kaplan–Meier survival curves (disease specific survival) comparing the high and low expression of the IL17A correlated 12 genes in HNSC patients (A-L).
